# Mitigation of Lethal Radiation Syndrome in Mice by Intramuscular Injection of 3D Cultured Adherent Human Placental Stromal Cells

**DOI:** 10.1371/journal.pone.0066549

**Published:** 2013-06-18

**Authors:** Elena Gaberman, Lena Pinzur, Lilia Levdansky, Maria Tsirlin, Nir Netzer, Zami Aberman, Raphael Gorodetsky

**Affiliations:** 1 Sharett Institute of Oncology, Hadassah - Hebrew University Medical Center, Jerusalem, Israel; 2 Pluristem Therapeutics Inc., Haifa, Israel; Leiden University Medical Center, Netherlands

## Abstract

Exposure to high lethal dose of ionizing radiation results in acute radiation syndrome with deleterious systemic effects to different organs. A primary target is the highly sensitive bone marrow and the hematopoietic system. In the current study C3H/HeN mice were total body irradiated by 7.7 Gy. Twenty four hrs and 5 days after irradiation 2×10^6^ cells from different preparations of human derived 3D expanded adherent placental stromal cells (PLX) were injected intramuscularly. Treatment with batches consisting of pure maternal cell preparations (PLX-Mat) increased the survival of the irradiated mice from ∼27% to 68% (P<0.001), while cell preparations with a mixture of maternal and fetal derived cells (PLX-RAD) increased the survival to ∼98% (P<0.0001). The dose modifying factor of this treatment for both 50% and 37% survival (DMF_50_ and DMF_37_) was∼1.23. Initiation of the more effective treatment with PLX-RAD injection could be delayed for up to 48 hrs after irradiation with similar effect. A delayed treatment by 72 hrs had lower, but still significantly effect (p<0.05). A faster recovery of the BM and improved reconstitution of all blood cell lineages in the PLX-RAD treated mice during the follow-up explains the increased survival of the cells treated irradiated mice. The number of CD45+/SCA1+ hematopoietic progenitor cells within the fast recovering population of nucleated BM cells in the irradiated mice was also elevated in the PLX-RAD treated mice. Our study suggests that IM treatment with PLX-RAD cells may serve as a highly effective “off the shelf” therapy to treat BM failure following total body exposure to high doses of radiation. The results suggest that similar treatments may be beneficial also for clinical conditions associated with severe BM aplasia and pancytopenia.

## Introduction

Radiation accidents such as those in Fukushima (2010), Goiânia, Brazil (1988), in Tokai-Mura, Japan (1999) and in higher scale in Chernobyl (1986) [1]–[4] serve as a warning sign of the hazards associated with potential future catastrophic nuclear events. Moreover, threats from exposure to high doses of radiation due to scenarios of criminal mega-terrorist events became more realistic in the recent years [5], [6]. In such events many individuals may be affected with no adequate estimation of the exact doses to which they were exposed. Easily available life saving treatments, which could be initiated successfully even a day or more after exposure and could be administered to large populations may be the only practical solution for such circumstances.

High dose exposure to lethal ionizing radiation results in deleterious systemic effects to different organs, including the reproductive system, the gastrointestinal (GI) tract, the liver, the skin, the kidneys, the central nervous system and the respiratory cardiovascular system [3], [7]–[13]. But the primary life threatening damage is inflicted to the most sensitive BM and hematopoietic system. The manifestation of the effects in acute responding tissues such as the GI, the epidermis and the BM is within a short period of a few days. But the effects could be delayed to many months in cases of sensitive late responding organs such as the lungs [14]. The critical life threatening complication is the acute hematopoietic syndrome with non-reversible destruction of the regenerative potential of the hematopoietic system [1], [2], [8], [12], [15]. Matched hematopoietic stem cells (HSC) transplantation may be a remedy of choice for the salvation of the eradicated BM, but it is not practical as an immediate treatment in an event associated with high dose exposure of many individuals. Other treatments could be based on growth factors, mainly granulocyte and granulocyte-macrophage colony stimulating factors (G-CSF and GM-CSF), which were approved as supportive treatment for BM regeneration following radiotherapy or chemotherapy and for enhancement of the engraftment of HSC in BM transplantation. G-CSF was proposed for emergency use as investigative new drug (IND) by the Centers for Disease Control and Prevention. Several other drugs and growth factors, as well as anti-inflammatory cytokines and chemokines are under investigation as radiation countermeasures [16]–[20]. The use of radical scavenger and DNA protecting agent WR2721 (Amifostine or Ethyol) [21], given before or very short time after radiation exposure was recently approved for the alleviation of clinical radiation symptoms [22]–[26]. Still none of those treatments could be considered as an ultimate life saving drug in cases of lethal high dose irradiation.

The critical effect on the GI following the exposure to doses of ∼ 4–10 Gy may also contribute to the BM failure due to a leakage of bacteria and related toxins from the sub-critically damaged guts to the circulation. This may severely challenge the immune system with possible aggravation of the lethal hematopoietic syndrome [1], [27].

Current concepts on radiation-induced insults are based on the assumption that an effective treatment modality should be administered immediately, within a few hours after radiation exposure to protect and prevent the death of the critically irradiated cells [1], [2], [15]. This leaves no practical solution for circumstances of a nuclear disaster with no accurate estimation of the high dose exposure, where the treatment may reach the affected individuals even days after a high dose exposure.

Cell therapies to reconstitute failing organs, aside from the hematopietic bone marrow (BM) stem cells transplantation, by indefinitely replacing the affected cells in most cases are not highly practical. The common understanding is that many such cell therapies are based on indirect regulatory mechanisms involving the secretion of growth factors and immune modulators by a wide variation of cell types [28]–[34]. Multipotent mesenchymal stromal cells from different sources, mainly from the BM, cord blood and adipose tissues were proposed for regenerative medicine. In most cases the limited therapeutic effect which was recorded was attributed to such indirect effects on the immune system and anti-inflammatory effects [35]–[42]. Moreover, the role of the “stemness” of the cells in the effect recorded in such cell therapies, relating to their ability for multipotent differentiation, is not straightforward [42]. Early works preceding the stem cells era suggested the local administration of young progenitor syngeneic cells to reverse radiation damage in organs such as skin [43], [44]. Later studies, investigating embryonic or adult mesenchymal stem cells for the reversal of radiation damage, could hardly show actual integration of these cells within the regenerating tissues [45]. Rather, an indirect support of the affected hematopoietic cells by secretion of modulating factors seemed to better explain the observed radioprotective effects [46]. The mechanism behind such “stem cells therapies” for the partial relief of the effects of chemotherapy and radiation treatments is not yet fully understood [47]–[50].

Placentas are an easily available source for high number of fast proliferating human cells from mesenchymal, endothelial and epithelial origin [51]. PLacenta eXpanded (PLX) cells, which were developed and manufactured by Pluristem Therapeutics, are adherent human-derived placental stromal cells lacking the expression of hematopoietic and endothelial specific cell markers, which are expanded in bioreactors in 3D conditions,. Pluristem presented their placental derived PLX-PAD cell as a potentially safe allogeneic cell therapy for the treatment of chronic conditions such as critical limb ischemia in peripheral artery disease [52] and suggested that they may also be employed for hematopoietic recovery [53]. The injected PLX cells are not stem cells by definition as they have limited differentiation potential. They are cleared away with time from the injected site and are not expected to be directly involved in replacing the affected cells in regenerating the failing tissues [52]. Their activity seems to derive from a transient indirect paracrine effects by secretion of pro-angiogenic and anti-inflammatory chemokines and cytokines before they are cleared away, with no apparent adverse effects and complications due to rejection [53].

In the current study we report on the use of 2 types of PLX cells for mitigation of acute radiation effects. We proposed to overcome the difficulties associated with IV stromal cells administration, where the cells are trapped mostly in the lungs immediately after their administration, as also previously reported by others [48], [54]. We expected a systemic endocrine secretion by these cells, not depending on their injection site, as long as it is highly vascularized, Therefore, we adopted the more convenient procedure of IM injections. This allows the easy delivery of higher cell numbers by injection in multiple time points. Such treatments were effective when they were initiated 24 hrs or more following total body irradiation, resulting in a significant mitigating of radiation induced complications.

## Materials and Methods

### PLX-Mat and PLX-RAD Source and Preparation

PLX (PLacental eXpanded) cells were produced and supplied by Pluristem Therapeutics Inc. (Haifa, Israel). The PLX are mesenchymal-like adherent stromal cells derived from full term placentas following Caesarean section. The PLX production is composed of two major steps of isolation and culturing of the adherent stromal cells in tissue culture flasks and a 3D growth phase on non-woven fiber made carriers in controlled bioreactors for their further 3D expansion. The cells were harvested and cryo-preserved in liquid nitrogen as an “off the shelf” allogeneic adult cell source product. Prior to their administration the cells were thawed, washed and suspended in Plasmalyte A solution (Baxter, Deerfield, IL, USA). Six different batches of cells were tested, 5 were analyzed by FISH XX:XY chromosomal assay to determine their cell ratio from maternal or fetal source. Three PLX batches of pure maternal cells were termed PLX-Mat and were similar to the cells called PLX-PAD in previous publications [52], [53], Two batches with a mixture of high proportion of fetal (male) offspring mixed with maternal cells were termed PLX-RAD. Additional PLX-RAD batch, in which the placenta was of a female offspring, was characterized as PLX-RAD based on the size of cells distribution with a sub-population of larger fetal cells and by their protein secretion profile, similar to the profile of the other 2 PLX-RAD batches.

### PLX-RAD Cells Membrane Markers Phenotype Assay

The PLX-RAD cells were labeled with PE conjugated mouse anti-human mAbs (Becton Dickinson, USA) against typical stromal markers, white blood cells and endothelial cells markers as described in the manufacturer’s manual. For CD markers phenotype determination the PLX cell batches were thawed, washed, re-suspended in FACS buffer containing phosphate buffered saline (PBS) with 2% fetal bovine serum (FBS), aliquoted for immunostaining (∼2×10^5^ cells/tube) and labeled with PE conjugated mouse anti-human mAbs (Becton Dickinson, USA) against typical stromal markers. Manufacturer recommended concentrations of FITC or PE conjugated mouse anti-human monoclonal antibodies (mAbs) (Becton Dickinson) of interest (mesenchymal markers and leukocyte markers or their isotypes) were added and cells were incubated at room temperature for 15 min. The cells were washed twice before flow cytometry analysis in a Beckman Coulter FC-500 system.

### In vitro Secretion Profile of PLX-RAD Cells

PLX-RAD cells were thawed in low glucose DMEM (Sigma Aldrich, US) with 10% fetal bovine serum (FBS) and 2 mM L-glutamine, and 0.5×10^6^ cells were seeded in 6 well-plates with 4 ml DMEM per well. Twenty four hrs later the medium was exchanged to EBM-2 (Lonza, Switzerland) and the plates were incubated for additional 24 hrs. The conditioned medium (CM) was collected and concentrated x10 using vivaspin 20 centrifugal concentrators (Sartorius, Stedim Biotech, France) at 4,500 RPM, for 1 hr at 4°C. Then the concentrated CM was analyzed using RayBio Human Cytokine Antibody Array, G-Series 4000 (RayBiotech, Inc., US) according to the manufacturer’s instructions. The array was then washed and incubated with biotinylated antibody and fluorescence-dye conjugated streptavidin. Signal detection was performed using a laser scanner (Axon GenePix by Molecular Devices, US). The fluorescence intensity values were normalized to positive controls of each measurement.

### Induction of IL-10 Secretion by LPS Activated Peripheral Blood Mononuclear Cells (PBMC)

PBMC were isolated from peripheral blood units purchased from Magen David Adom, central blood bank, Tel-Hashomer, Israel, The separation was done by centrifugation for 30 min at 25°C in 2,000 RPM in Lymphoprep (1.077 g/ml; Fresenius Kabi, Germany). The PBMC ring created above the lymphoprep layer was collected, washed with Hanks Buffer (Gibco, US) and the resulting PBMCs were aliquoted (10×10^6^ cells/ml/cryo-vial) and cryopreserved in liquid nitrogen. PLX-RAD cells were thawed into RPMI 1640 (Biological Industries, Israel), completed with FBS 10%, L-Glutamine 2 mM, and seeded in 96 wells plate in replicates of 6 (100,000 cell/50 µl RPMI/well). Following plating, the cells were incubated for 24 h (37°C, 5% CO_2_). After 24 hrs PBMCs were thawed in RPMI medium and seeded in the same plate with or without lipopolysaccharide (LPS; Sigma Aldrich, US), at final concentration of 7.5 µg/ml, to create a co-culture or appropriate controls. Then, the plate was incubated at 37°C, 5% CO_2_. Five hrs later, CM were collected from the wells and submitted to IL-10 concentration quantification using sandwich ELISA (Quantikine ELISA kit by R&D systems, US) according to the manufacturer instructions.

### PLX Cells Administration

The PLX cell batches used were defrosted in Plasmalyte A (Baxter, Deerfield, IL, USA) +5% clinical grade human albumin (Biotest. Pharma GmbH, Dreieich, Germany), this resulted by 80–95% cell survival as assayed by trypan blue excretion assay. The cells were washed and re-suspended to 20×10^6^/ml in Plasmalyte A. The dose given in any injection contained 2×10^6^ cells in 100 µl Plasmalyte A medium. The acquired cell suspension was delivered by 25 µl injections to 2 sites of the thigh muscle of each hind extremity (total of 100 µl containing 2×10^6^ cells). The control irradiated group was injected similarly with the same volume (100 µl) of Plasmalyte A (vehicle-control).

### Mice Maintenance and Follow Up

C3H/HEN-HSD male mice, 8–9 weeks old were purchased from Harlan Laboratories, Rehovot, Israel. The mice weighing ∼25–27 gr in average were housed for at least an additional week for acclimation in specific pathogen free (SPF) facility before their inclusion in the experiments, The studies were approved by the Institutional Animal Welfare Committee of the Hebrew University of Jerusalem #MD-12-13296-4 (NIH approval number OPRR-A01-5011).

### Total Body Irradiation of the Mice and Survival Assay

The mice were immobilized within a sterilized irradiation in sterilized jig. They were restricted by the height of the jig to assure uniform and accurate dose exposure. Irradiation was done by a clinical 6–18 MeV LINAC (Varian, Medical Systems, CA, USA). The dose given by this setup was accurately calibrated and calculated by thermoluminescent dosimeter (TLD) and ionizing chambers by clinical physicist-dosimetrists that routinely monitor these clinical devices.

During the period of the experiment the mice were kept and monitored in SPF conditions. The animals were inspected daily for survival and weighted at least 3 times a week.

### Differential Analyses of Peripheral Blood, Whole BM Cell Counts and FACS Analyses of the Nucleated BM Cells

At different time points of interest and at the end of the experiments, typically at day 23, the mice were sacrified. Prior to that, they were deeply anaesthetized and ∼300 µl of blood was sampled from retro-orbital sinus for differential cell counting by COULTER® LH-750A Hematology Analyzer, Beckman Coulter, Inc (Nyon, Switzerland). The total counts of red blood cells (RBC) and related parameters of hematocrit (HCT) and hemoglobin, total nucleated white blood cells (WBC) and platelets (Plt) were determined.

Following the blood extraction the animals were sacrificed and total BM cells were harvested from the both femurs and tibias as follows: the extremities of the bones were cut off and all the BM plugs were fully flushed out of the bone cavities with PBS+2% FCS. The BM was suspended in the PBS+FCS and counted by hematocytometer. Then, BM cells were stained for CD45 and SCA-1 markers as follows: the cells harvested from each mouse were added to FACS tubes. The tubes were incubated with rat anti-mouse CD16/32 mAb (2 µl/10^6^ cells/100 ul FACS buffer/tube) for 15 min at RT and then stained using FITC-conjugated rat anti mouse CD45 mAb (0.5 µl/10^6^ cells/100 µl FACS buffer/tube) and PE-conjugated rat anti-mouse SCA-1 mAb (2.5 µl/10^6^ cells/100 ul FACS buffer/tube). Isotype controls were made with FITC-conjugated Rat IgG2b, κ isotype controls (0.5 µl/10^6^ cells/100 ul FACS buffer/tube) and PE-conjugated Rat IgG2a, κ mAb (2.5 µl/10^6^ cells/100 µl FACS buffer per tube). After 15 min of incubation at RT in the dark 2 ml of RBC lysing buffer were added to each sample. The cells were incubated at RT for 10–15 min, protected from light, centrifuged and then supernatant was aspirated without disturbing the pellet. The remaining cells were washed twice in FACS buffer and then analyzed by flow-cytometry (FC-500 Coulter System, Beckman CA). All the reagents used for BM analysis by flow-cytometry were purchased from Biolegend® (USA).

### Histology

For regular histology intestines, heart, liver and lungs were excised and fixed in 4% formaldehyde solution. Bone sections were performed on 2 days decalcified bones in 1∶1 mixed 8% formic acid and 8% hydrochloric acid. The samples were then washed in running water for 5 hrs before embedding in paraffin. The slides were stained by hematoxylin and eosin (H&E). When applicable, some slides of muscles were only deparaffinized and prepared for fluorescence microscopy of the remaining stained cells in the sections.

### Fluorescence Microscopy of Stained Live-cells within Freshly Excised Unprocessed Tissues

PLX-RAD cells were stained in suspension for 15 min at 37°C with carboxyfluorescein diacetate succinimidyl ester (CFSE) probe (CellTrace™ CFSE Cell Proliferation Kit, Invitrogen, Oregon, USA) according to manufacturer instructions. The final concentration of 5 µM for CFSE was chosen based on in-vitro studies in which this concentration showed no toxicity while yielding high fluorescence intensity which could be detected even after 3–4 cell divisions in cell culture for 2 weeks, as previously shown [55]. Following CFSE staining the cells were washed with fresh medium and incubated for 30 min in 37°C according to the manufacturer instructions. The CFSE underwent acetate hydrolysis which left the stable fluorescence dye trapped in the cells without affecting their activity. Before IM injection the CFSE stained cells were washed with PBS and suspended in Plasmalyte A in a concentration of 20×10^6^ cells/ml (100 µl/mouse = 2×10^6^ cells). At different time points of interest after the IM administration, lungs, heart, kidneys, spleen and leg muscles from the injection site were excised and viewed by direct fluorescence microscopy. By this procedure an unprocessed slice of the freshly excised whole organ, ∼1–2 mm thick, was cut off, rinsed in normal saline and placed slightly pressed between 2 microscope slides. The unprocessed sample was then viewed by both light and fluorescence microscopy so that the stained cells distribution in the bulk tissue could be clearly recorded.

### Dose Modification Factor (DMF) Evaluation

The DMF is the ratio between the doses given in treated relative to untreated animals to reach the same survival effect. To test the DMF of PLX-RAD, several groups of animals were exposed to escalating doses of total body irradiation with or without IM administered PLX-RAD treatment on days 1 and 5 after irradiation The endpoints in our study were the ratio between the doses that resulted with 50% or 37% survival (LD_50_ or LD_37_, respectively) in both the experimental and control arms.

### Statistics

Statistical analyses for the comparison between 2 groups of interest and the control group were done by Student’s t-tests, assuming equal variances, with one-way analysis of variance and *post-hoc* corrections for multiple group comparison when applicable.

The significance of the difference between the survival curves were analyzed by both a Log-Rank and Wilcoxon -2Log (LR) tests of the Kaplan-Meier survival curves, with *post-hoc* correction was applied for analyses of the duration of survival and the endpoint survival rate following the different treatments tested.

## Results

### PLX-RAD Cells: Phenotypic Markers and Specific Secretion Profile

**Figure 1:**
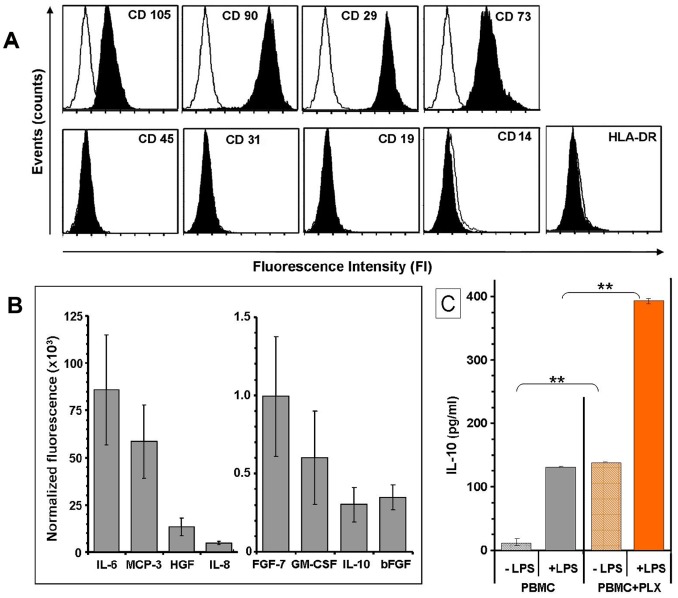
In-vitro characterization of the PLX-RAD cells. A. Representative FACS profile of cell surface markers of PLX-RAD cells batches used in the current study. The PLX-RAD cell population could be defined as having typical human mesenchymal markers (CD 105, 29, 73 and 90), with no contamination of cells expressing WBC markers (CD 45, 19, 14 and HLA-DR) or endothelial cells marker (CD31). B. Secretion profile of protein of interest by PLX-RAD cells in vitro. The protein secretion composition of PLX-RAD cell batches (n = 3) after 24 hrs incubation in EBM-2, using a protein array. Levels of IL-6, MCP-3, HGF, IL-8, FGF-7, GM-CSF, IL-10 and bFGF in the conditioned medium are shown. C. Induction of IL-10 secretion by PBMC in the presence of PLX-RAD. The PLX-RAD cells were co-cultured with PBMC with or without LPS, and IL-10 release in the media was measured by sandwich ELISA plate. PLX-RAD alone increased the IL-10 secretion significantly and induction with LPS further significantly increased the PLX-RAD effect (** = p<0.01).

The PLX-mat and PLX-RAD batches tested in this study had FACS profile of surface markers (Fig. 1A) positive to CD 105, 29, 73 and 90, with no expression of either blood cells markers such as CD 45, 19, 14 and HLA-DR or endothelial cells marker (CD31). The typical in-vitro cytokines secretion profile of PLX-RAD batches is shown in Fig. 1B. These cells had a higher secretion of IL6, MCP3, FGF7 and GM-CSF and IL-10 than PLX-Mat (data not shown). The biological activity of the cells was also assayed *in-vitro* for their modulation of cytokines mediated inflammatory responses (Fig. 1C). *In-vitro* Stimulation of PBMCs with LPS increased significantly their IL-10 secretion. In the presence of PLX-RAD the IL-10 secretion was much further elevated (p<0.01). These results indicate that adding PLX-RAD cells to PBMC induce elevation of the secretion of the anti-inflammatory factor IL-10 both in non-stimulated or LPS activated PBMC.

### Effect of IM Delivery of Different PLX Cell Batches on Irradiated Mice

The C3H/HeN mice used in this study are relatively radioresistant to total body irradiation, as compared to strains such as commonly used BALB/C. All the C3H/HeN mice survived 6 Gy irradiation and only 20% of them survived exposure to a higher dose of 8.5 Gy (data not shown). Exposure to a dose of 7.7 Gy resulted in ∼30% survival. This dose was chosen for the experiments on the effect of PLX cells injection on mice survival since it allowed the survival of small, but still sufficient number of the Vehicle control treated mice out of the numerous animals included in this group to follow-up their blood profile and BM cell counts at the end of the experiments.

**Figure 2:**
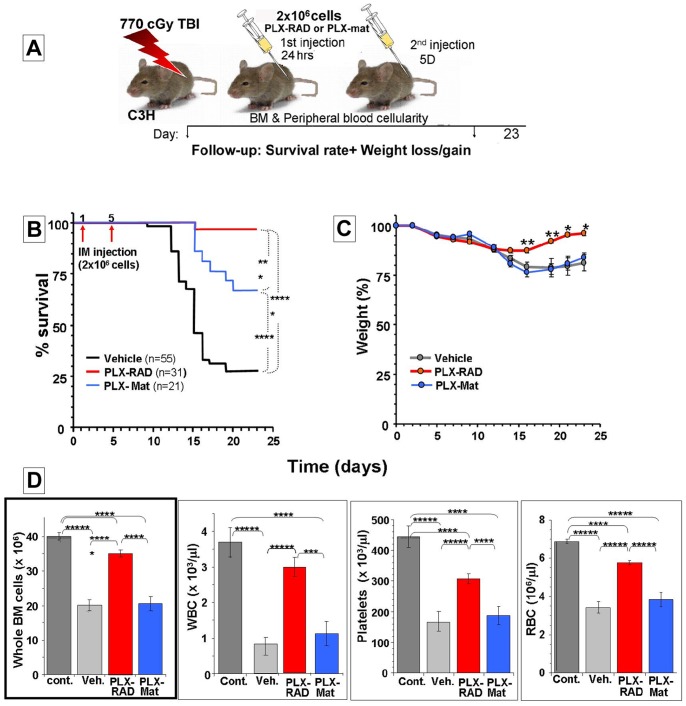
The effect of IM PLX-Mat and PLX-RAD injection on the survival of 7.7 Gy total body irradiated mice. The setup of the experiment is described in (A). 2×10^6^ PLX-RAD or PLX-Mat cells were injected IM twice, 1^st^ injection delivered 24 hrs and 2^nd^ injection on day 5 after irradiation. The follow-up of mice survival is shown in (B). Weight changes for the group of IM injected mice at day 1 and 5 after irradiation are presented in (C). The BM and blood counts of the surviving animal at the end of the experiment at day 23 are presented in (D). Significance: * = P<0.05, ** = P<0.01*** = P<0.005, **** = P<0.001, ***** = P<0.0001.

In a set of radiation survival experiments PLX-RAD or PLX-Mat cells (2×10^6^) were injected IM to the pre-irradiated mice. The experimental protocol and plan are sketched in Fig. 2A. In preliminary probing experiments lower numbers of PLX-mat cells were delivered in a single IM injection with a limited effect (not shown). Therefore, in further experiments a higher number of 2×10^6^ cells were injected in 2 time points, typically on day 1 and 5 after irradiation. This treatment schedule yielded the optimal results which are presented in this study. The data were based on merging repeated individual experiments of 9–12 mice per arm in which each separate experiment yielded similar results for the same arms. The irradiated control mice were injected twice with the Vehicle with no cells. In one arm of the experiments 2×10^6^ PLX-RAD cells were delivered IM twice to 31 mice, 24 hrs and 5 days following irradiation by 7.7 Gy. In another arm 21 mice were treated with PLX-Mat. None of the IM treated animals had any signs of stress after either the 1^st^ or the 2^nd^ cells injection. The survival curves of the 7.7 Gy total body irradiated animals subjected to IM administration of PLX-Mat and PLX-RAD or injected with vehicle controls are shown in Fig. 2B. Only 27% of the irradiated vehicle treated mice survived beyond day 16, while the survival of the mice treated with IM PLX-Mat cells reached 67%. In mice treated with PLX-RAD cells 97% survived and fully recovered from the radiation insult. This difference in the survival rate was highly significant in comparison to both the control Vehicle treated group (p<0.0001) and the PLX-Mat group (p<0.005). The PLX-RAD treated mice regained their weight significantly faster than the PLX-Mat and survivors of the vehicle control treatment (Fig. 2C). In none of the arms was a death recorded beyond day 18, therefore the experiments were typically terminated as early as day 23, at which stage the recovery of the survivors in the different arms was obvious and the mice were in the process of regaining their lost weight. Only the PLX-RAD treated mice fully regained their initial weight at this stage.

The early termination of the experiments at day 23 after irradiation allowed monitoring the difference in the blood and BM profile between the survivors of the experimental groups tested, before these differences faded. The data on the whole BM, platelets, RBC and WBC counts at the termination of the experiments are presented in Fig. 2D. While the survivors of the PLX-Mat had only minor non-significant better BM and blood cells profile than the vehicle controls, the PLX-RAD treated animals, which almost all survived, had very significant elevation of all these parameters relative to both the PLX-Mat treated mice and the few strongest surviving vehicle control treated mice.

Based on the significant advantage of the PLX-RAD treatment, the further detailed studies which are described below were performed only with these cell preparations.

**Figure 3:**
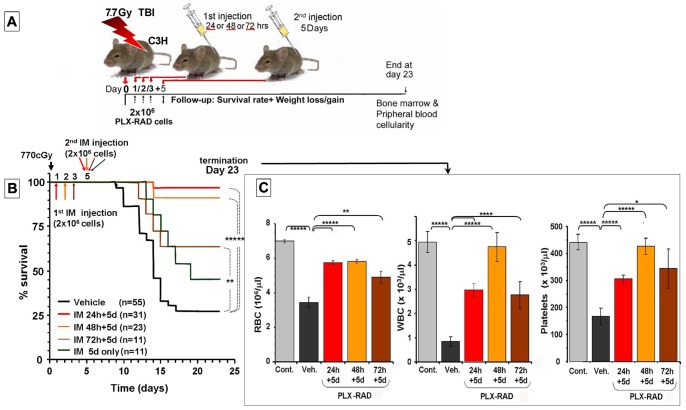
The effect of IM PLX-RAD injection on the survival of 7.7 Gy total body irradiated mice. The setup of the experiment arms is described in (A). PLX-RAD cells (2×10^6^) were injected IM twice, 1^st^ injection was done 24 or 48 or 72 hrs and 2^nd^ injection was done on day 5 after irradiation. In another arm only a single injection on day 5 was given. The data presented are based on merged separate experimental repetitions. The total number of animals in each arm is shown in the legend. The follow-up of mice survival between the different schedules of treatment is shown in (B). The BM and blood cells profile of the surviving mice at the end of the experiments on day 23 is shown in (C). Significance: * = P<0.05, ** = P<0.01, **** = P<0.001, ***** = P<0.0001.

To test the time limits of injection schedules to achieve the desired effect of radiation protection by IM injected PLX-RAD cells, the 1^st^ cell administration was delayed from 24 to 48 or 72 hrs. The experimental plan is shown in Fig. 3A. Delaying the initiation of the treatment for 48 hrs after irradiation had almost similar effect (91% survival) to a delay of 24 hrs (98% survival). Further delaying the 1^st^ injection to 72 hrs still showed a significant residual effect of the PLX-RAD cells treatment, with 55% survival. But when the first injection was skipped and given only on day 5 the effect of the injected cells was no longer significant (Fig. 3B). The blood counts on day 23 of the mice treated 24 hrs after irradiation by the protocol of IM injections of PLX-RAD were compared with the arms where the first injection was delayed by 48 and 72 hrs (Fig. 3C). Even if the first IM PLX-RAD injection was delayed by as much as 72 hrs after irradiation, the recovery of the different blood cell types was still significantly higher than the data recorded from the mice treated with vehicle control.

**Figure 4:**
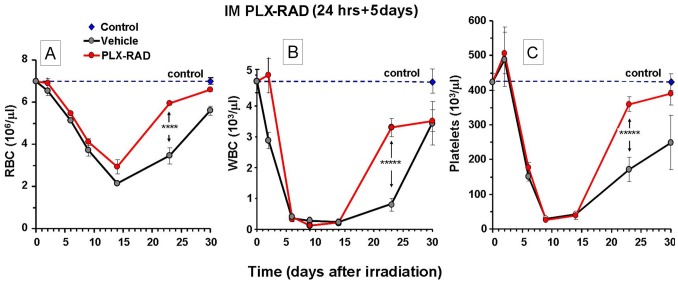
Time dependent alterations of the profile of the 3 peripheral blood cell lineages following irradiation and PLX-RAD treatment. Mice were injected with PLX-RAD on day 1 and 5 following 7.7 Gy irradiation. The mice were bled and sacrificed on days 2, 6, 9, 14, 23 and 30 after irradiation. RBC (A), WBC (B) and Platelets (C) were counted in peripheral blood. The data are based on at least 8 mice which were included for each group for the different rime points. For day 23 the data are based on 31 PLX-RAD treated and 55 vehicle treated mice (from repeated experiments). The recovery on day 23 of peripheral blood cells counts of the surviving mice subjected to the different tested arms with different timing of the first IM injections is presented. The more affected animals died before the end of the experiment, mostly in the group of vehicle treated controls, leaving only the few survivors on which the relevant data are based. The profile of the WBC, platelets and RBC are presented. Significance: * = P<0.05, ** = P<0.01, **** = P<0.001, ***** = P<0.0001.

The combined data of blood counts from the experiments which were terminated at different time points after 7.7 Gy irradiation and PLX-RAD treatment at day 1 and 5 days are given in Fig. 4. The highest number of animals were examined on day 23, when most of the experiments were terminated and blood samples and BM cells were collected (see Fig. 2). The data was combined with other time points, each based on data from 8–10 mice, which were sacrificed at days 2, 6, 9, 14 and 30. At day 23 the PLX-RAD treated mice had significantly higher RBC, WBC and platelets counts relative to the fewer surviving mice treated with vehicle control group. The data on RBC were similar to the data on the hemoglobin level and the hematocrit. Therefore, only the RBC counts are presented. On day 30 RBC and platelets counts were almost fully recovered to normal levels in the group treated with PLX-RAD cells, while the WBC count was similar to the few survivors of the vehicle control group.

### Localizing and Time of Residence of the Injected PLX-RAD Cells by IM Injection

**Figure 5:**
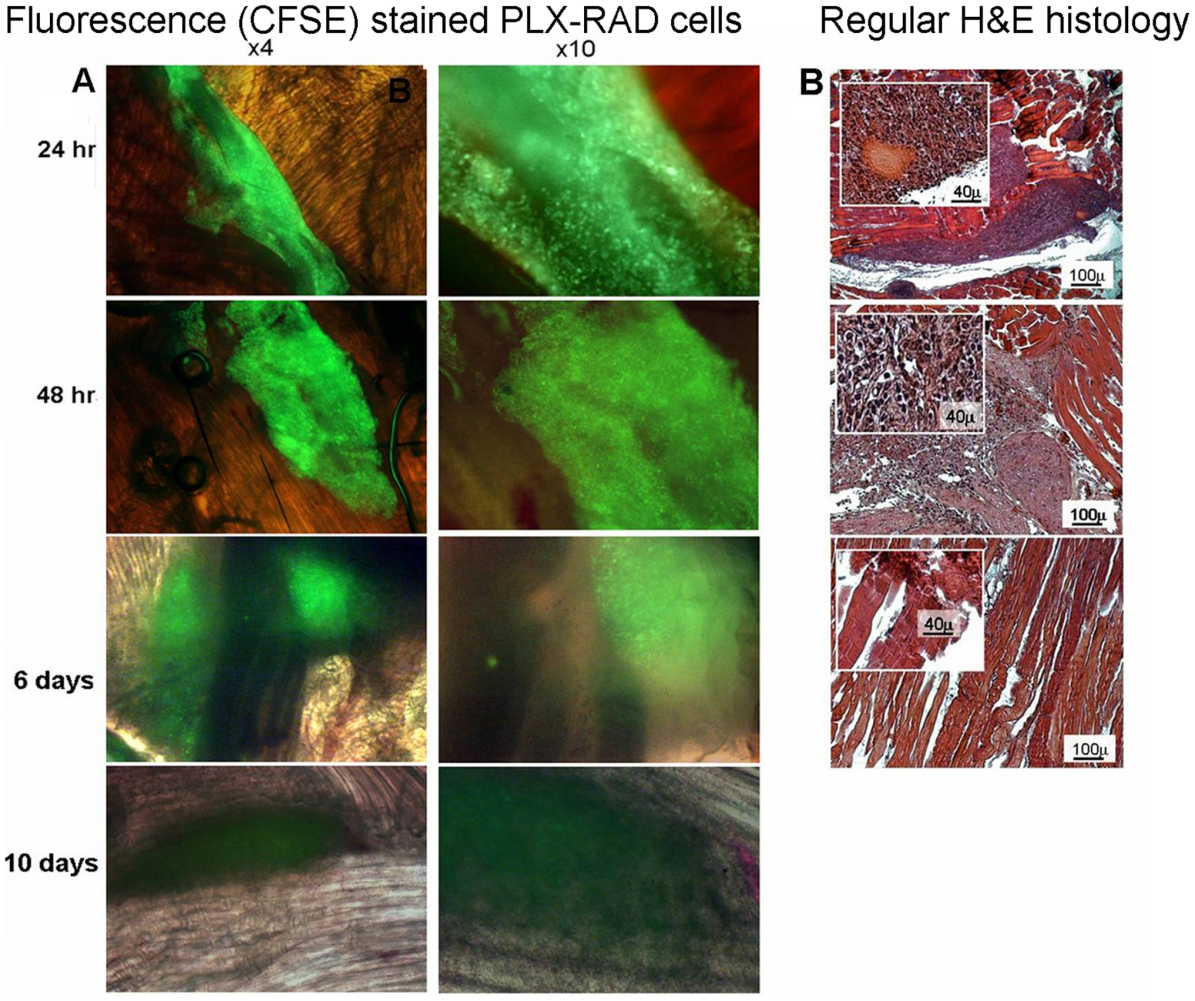
The presence of PLX-RAD cells in the injection site. (A) 2×10^6^ CFSE labeled cells PLX-RAD cells were injected IM to 7.7 Gy irradiated mice. Direct fluorescence microscopy images with dim white light background (as described in methods and materials) at different magnifications are presented. The injected cells were observed only within the injection site in the freshly excised intact muscle. Direct fluorescence microscopy showed slow clearance of the cells within 6–10 days. (B) Regular H&E stained histological sections of samples taken 24, 48 hrs and 6 days after injection. An inset in each of the H&E histology shows a higher magnification of the area within the muscles where the injected cells resided are clearly seen.

In an attempt to trace where the PLX-RAD cells are localized following IM treatment, 2×10^6^ PLX-RAD cells pre-stained with CFSE were injected IM. The mice were sacrificed at different time points after cells administration and their major organs were dissected and examined by direct fluorescence microscopy, as described in the section of the methods. The combined fluorescence and dim light microscopy enabled to see cells residence in the unprocessed fresh tissues. After their IM injection and during the whole follow-up, the CFSE stained PLX-RAD cells were detected restricted only within the site where they were initially injected (Fig. 5A). No stained cells were traced in any of the dissected organs, including heart, lungs, spleen, kidneys and BM during this follow-up, which ruled our their migration to other organs. The cell aggregates in the injected muscles were clearly observed for up to day 6. By day 10 after injection only faint residual traces of the cells remained. Samples of the muscle tissue, in which the fluorescent cells in the injected area were identified, were dissected and prepared for regular H&E histology. The results are presented for days 1, 2 & 6 in Fig. 5B. At days 1 and 2 after IM injection the H&E histological sections of the injected muscles revealed the large aggregates of the injected cells trapped within the intact muscle, as also seen by the direct fluorescence scanning of the intact muscle before their fixation. No signs of adverse effects or any apparent local inflammatory process was seen till the fluorescence of the cells was not apparent anymore.

### Effect of the PLX-RAD Administration on Radiation Induced BM Damages

**Figure 6:**
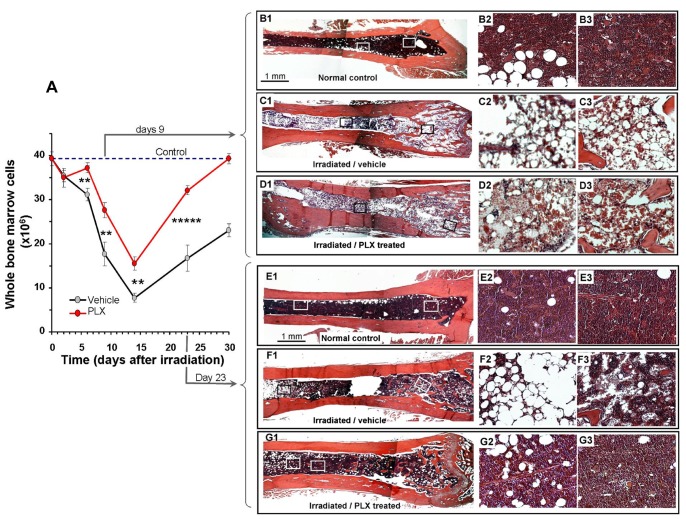
Kinetics of whole BM cellularity in mice treated on days 1 and 5 after irradiation with IM injections of PLX-RAD or vehicle controls. The whole BM cells were extracted from the tibias and femurs of the surviving treated animals from the 2 experimental arms on days 2, 6, 9, 14 and 23 after irradiation. Each group consisted of 8–10 mice. A clear advantage of the total cell number in the PLX-RAD injected mice was recorded along the follow up and was most significant from day 9 onward (A). In parallel samples of histological sections of decalcified bones from PLX-RAD and vehicle treated animals relative to bones from non-irradiated mice on day 9 and 23 are presented (B-G), further demonstrating the advantage of the PLX-RAD treatment in this critical time points. The histological bone sections are presented from day 9 for a non-irradiated control (B1 with magnified frames in B2 and B3), from vehicle controls (C1 with magnified frames in C2 and C3), and PLX-RAD treated mice (D1 with magnified frames in D2 and D3). The histological sections show only marginal better cellularity in the PLX-RAD treated mice on day 9 relative to vehicle controls. On day 23 after irradiation the difference was maximal, as also reflected in the relevant histological slides (F_1–3_ and G_1–3_) similar to the cellularity observed in non-irradiated control mice (E_1–3_). Significance: ** = p<0.01, ***** = p<0.0001.

**Figure 7:**
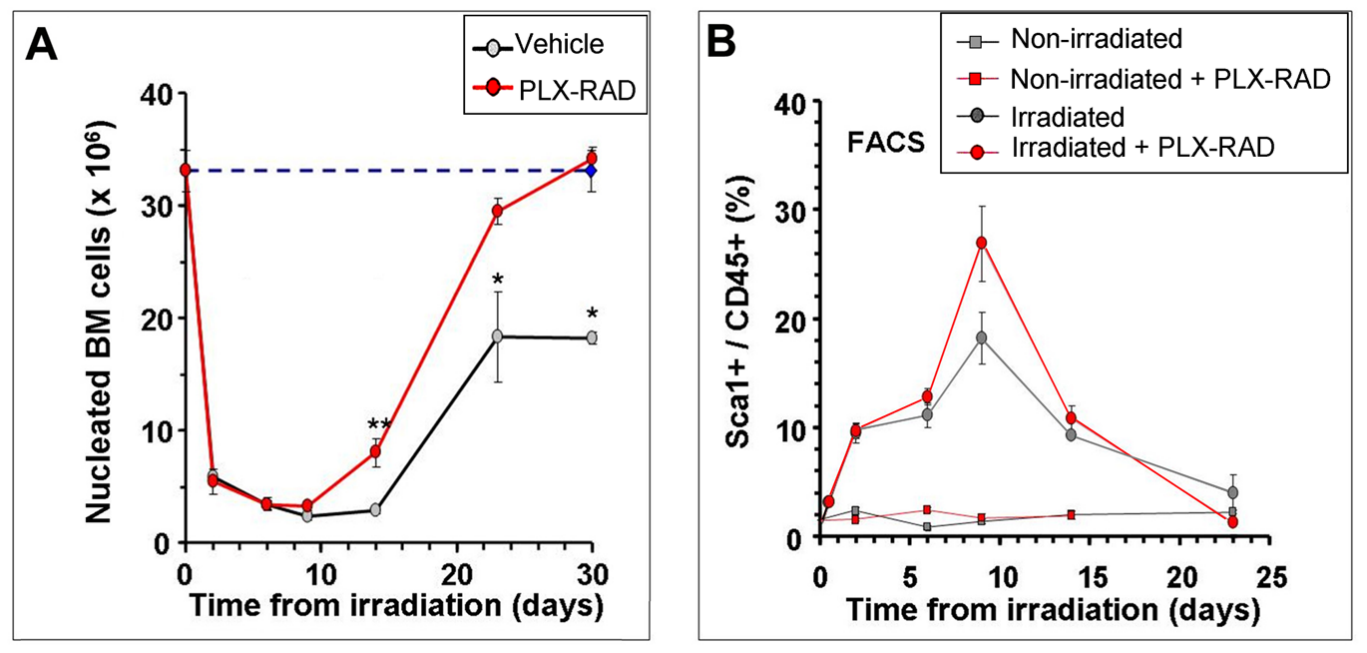
The effect of IM injection of PLX-RAD cells on days 1 and 5 after radiation on the number of nucleated cells from whole BM and the ratio of progenitors within this cell population. (A) Up to day 9 after irradiation, the number of the nucleated BM cells decreased sharply in both arms, followed by a faster gain in the number of total nucleated BM cells in the PLX-RAD treated animals, as compared to the vehicle controls. By day 30 a full recovery of the number of nucleated BM cells was recorded only in the group of irradiated PLX-RAD treated mice. (B) FACS analysis of the kinetics of changes in the % of CD45^+^/Sca-1^+^ of the nucleated cells (representing the progenitor cells population) was tested in both arms of mice irradiated by 7.7 Gy, PLX-RAD or vehicle control treated. Maximal increase in the proportion of these cells due to the radiation exposure was recorded on day 9 in both groups, before the onset of the critical phase of the hematopoietic syndrome. Significance: * = P<0.05, ** = P<0.01.

The effect of IM PLX-RAD cells injection 1 and 5 days after radiation on the total number of nucleated BM cells was evaluated. The total counts of BM cells harvested from the major long bones of both hind limbs and followed up at different time points are shown in Fig. 6A. Histological preparations of decalcified sections of long bones of the hind limbs are shown in Fig. 6B–G. On day 9 following irradiation, before the onsets of the severe lethal phase of the acute hematopoietic syndrome, all the mice from both groups were still alive. Radiation induced effect on the total number of BM cells was observed in both groups of irradiated mice injected with PLX-RAD cells or with vehicle controls. A significant difference in the total number of BM cells was observed in the PLX-RAD treated mice relative to the mice treated with the vehicle control (Fig. 6A). This was also visually apparent in histological sections of the bones (Fig. 6B_1–3_–D_1–3_). From day 14 onward the cell number of the BM increased in the survivors of both groups, but a very significantly faster recovery was seen in the group of PLX-RAD treated mice which reached a maximal difference at day 23 following irradiation, when most of the animals were sacrificed (Fig. 6A). In mice that were examined on day 30 the BM was fully recovered only in the group of PLX-RAD treated mice, which almost all survived the radiation exposure, and not in the few survivors of the group treated by the vehicle only, as also shown in the histological sections of the long bones (Fig. 6A, F_1–3_ and G_1–3_ relative to E_1–3_). These data are supported by the count of only the nucleated cells which were collected from the whole BM and further tested for progenitor surface markers by FACS analysis (Fig. 7). The reduction of the number of nucleated cells was almost similar in both PLX-RAD and vehicle treated groups of irradiated mice on days 3, 6 and 9, before the onset of the hematopoietic syndrome (Fig. 7A). But from day 14 onwards the PLX-RAD treated animals, relative to those treated with vehicle control, showed much faster regeneration and increase in the number of nucleated cells, till a full recovery was observed at day 30. Treatment with PLX-RAD cells in non-irradiated controls also increased the number of whole BM and nucleated BM cells above the normal level for up to ∼10% within 14 days after the 1^st^ PLX-RAD injection (Not shown). The faster reconstitution of the BM in the mice treated by PLX-RAD cells may be explained by enhanced proliferation of the subpopulation of progenitor cells within the nucleated BM cells which are positive to both CD45^+^ and Sca-1^+^ as seen by FACS analysis. Combining the higher number of total nucleated cells with the elevation of the proportion of the progenitors in this cell population may explain the much faster life saving recovery of the BM from day 9 onwards in the mice treated with PLX-RAD cells.

### Dose Modification Factor by PLX-RAD Treatment

**Figure 8:**
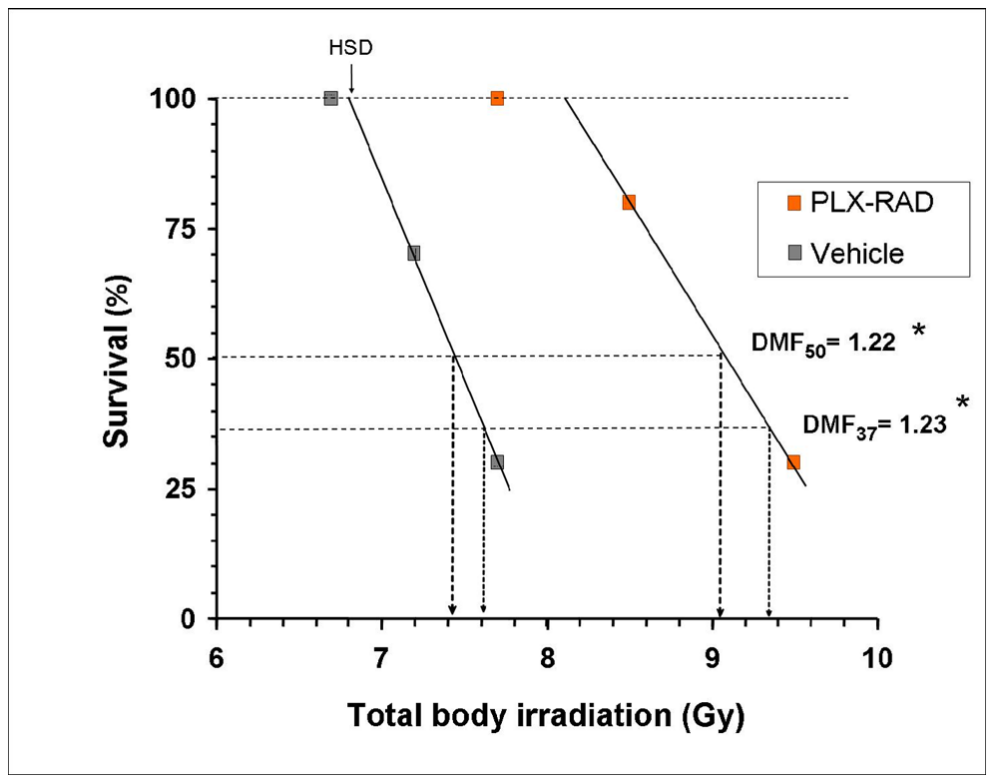
Evaluation of the dose modifying factor (DMF) of IM treatment with PLX-RAD. The ratio between the total dose needed to reach 50% survival rate of animals treated with PLX-RAD cells (9.05 Gy) relative to the dose needed to yield the same effect in the controls (7.42 Gy) was calculated based on the survival of irradiated mice exposed to different doses of radiation +/− IM injection of 2×10^6^ PLX cells at days 1 and 5 after irradiation. A DMF of 1.22 was recorded. The highest supported total body irradiation dose (HSD) which still yields 100% survival in the irradiated controls can be extrapolated from this Figure to be ∼6.8 Gy.

The results of DMF calculations are presented in several groups of animals were exposed to escalating doses of total body irradiation with or without IM administered PLX-RAD treatment on days 1 and 5 after irradiation (Fig. 8). This value was found to be significant for both DMF_50_ and DMF_37_ (1.22 and 1.23 respectively, p<0.05). We also extrapolated the value of the highest dose exposure in which the animals could be expected to survive irradiation, as we termed it “highest sublethal dose” (HSD) which is expected to increase in the protected animals. This parameter could be extrapolated from our data to yield a value of ∼7.8 Gy for the PLX-RAD treated mice relative to a value of ∼6.8 Gy for the irradiated vehicle controls.

## Discussion

Remedies for the accidental exposure of individuals, as well as mass populations, to high doses of ionizing radiation are scarce. Adequate practical clinical treatments are expected to be safe with simple route of administration which could be available as on “off the shelf” product for fast delivery. Moreover, practical treatments should be effective even if given in a significant delay from the radiation exposure, to allow the shipment of the cells to the site of the accident [15], [47], [49], [50].

Recent studies suggested the administration of syngeneic or allogeneic mesenchymal stem cells (MSC) from different syngeneic or allogeneic sources [50], [56]–[58], as well as myeloid precursors [59] to enhance the recovery of damaged BM and for mitigation of radiation effects. The mechanism of action of such treatments and their safety are being questioned. The generally practiced IV cell delivery was probably based on the expectations that the cells should target somehow the BM niche in order to be effective, similar to the outcome of HSC transplantation. Nevertheless, as shown by many studies and by our experience, IV injection of stromal cells results with their immediate full detainment within the capillaries of the lungs, where they reside for a limited time interval with no significant targeting to other organs before being cleared away [42], [57], [60].

The cell therapy which we proposed with the use of different batches of stromal placental cell preparations (PLX-Mat and PLX-RAD) seem to have an indirect systemic effects, probably based on their ability to secrete adequate modulating cytokines and chemokines to the circulation. We also showed in vitro that upon their exposure to inflammation associated factors such as TNFα the PLX-RAD cells secrete high levels of immune modulators such as IL10. We expected that *in vivo*, since the effect of the cells is indirect, the cells could mitigate the radiation effect when administered into any highly vascularized site, such as the large muscles, and not necessarily systemically. Such mode of delivery allows the injection of high cell numbers by repeated injections, with no apparent adverse effects and temporary stress which we have observed with IV administration. In such preliminary experiments the cells could mitigate the radiation effect also when administered IV a day after irradiation (not shown), but the injected cells induced a significant stress immediately following their administration when they were trapped in the lungs. Therefore, we eventually adopted the IM administration as a safer effective treatment. The injected cells were shown to reside for at least 6 days after their administration in the injected site. The fact that no adverse effect or inflammatory reaction was observed in the injected muscles suggests that a non-inflammatory process, such as apoptosis, may be involved in the fading of the cells with time, as previously proposed for cells trapped in organs such as the lungs following IV injection [42], [49], [52].

The survival rate of the control group of the mice exposed to total body irradiation of 7.7 Gy and then treated IM by vehicle control administration was only ∼27%. These values were raised to 67%, and 97% following IM treatment with PLX-Mat and PLX-RAD cells, respectively. Therefore the main following studies were focused on the effect of the more potent PLX-RAD cells. In the conditions tested IM injection of PLX-RAD cells 24 hrs after radiation exposure, followed by 2^nd^ injection on day 5, yielded best mitigation of the radiation effects. The success of the IM treatment suggests that the observed effect was systemic, and not by homing and direct participation of the injected cells in the reconstruction of the radiation damaged BM niche. This indirect effect of the treatment allowed overcoming the BM syndrome of the treated mice, with almost full recovery of their hematopoietic systems within the 23 to 30 days of follow-up.

The time window for the initialization of the treatment after exposure to high dose of radiation is critical for practical applications. Delaying the initiation of the PLX-RAD treatment from 24 hrs to 48 hrs still yielded a full effect while a further delay for 72 hrs showed much lower (but still significant) protective effect. A direct protection of the progenitor cells from radiation by enhancement of DNA repair processes can occur only within a limited time interval of up to 6–24 hrs after radiation exposure. Therefore, this kind of direct cell protection cannot explain our data [42], [57], [60]. Rather the systemically delivered induction of the enhancement of the rate of proliferation of the residual surviving HSC in the BM, which otherwise are too few to save the animal in the critical phase of BM cell depletion seems to be a more plausible explanation for this effect. This is supported by the faster elevation of BM cellularity and the 3 peripheral blood cell lineages in the PLX-RAD treated mice after theiir initial critical radiation induced depletion.

The different blood cell lineages are depleted from the circulation a few days after high dose irradiation due to the inability of the heavily irradiated HSC in the BM to replace these circulating cells. The subsequent critical point of BM crisis after high dose irradiation is reached from days 7–9 onwards. We suggest that the PLX treatments effectively boosted the proliferation of the surviving HSC within this critical time window. Therefore, the treatment could be delayed by one or two days after irradiation and still be highly effective and save the irradiated mice before they face the critical phase of lethal pancytopenia. The suggested explanation for the effect of PLX-RAD treatment, which conforms with our data, is that these cells secrete stimulating factors for the induction of faster proliferation of the very few residual HSC which survive the lethal irradiation, as shown for the kinetics of nucleated BM cells and WBC on days 6–9 after irradiation. We found that the initial recovery of the BM of the mice treated with PLX-RAD cells coincided with the fast elevation of the proportion of CD45+/SCA-1+ progenitor cells in residual nucleated BM cells, in comparison to the irradiated vehicle controls. The combination of enhanced proliferation rate of the nucleated BM cells and the elevated fraction of the progenitors in this population allows the expedited release of much higher number of functional blood cell leneages to the circulation, just before the onset of the severe acute radiation-induced BM syndrome. Further investigation may elucidate why the mixture of the maternal and fetal stromal cells of the PLX-RAD batches mitigated consistently better the radiation effect than the pure maternal PLX-Mat cells.

Both DMF_37_ and DMF_50_ for PLX-RAD treatment were found to be ∼ 1.23 (Fig. 8). Since the animals were relatively radioresistant with a threshold of mortality (HSD) as high as 6.8, the DMF value may underestimate the mitigation effect of the PLX-RAD cells, which allowed the delivery of extra 1.5 Gy in the PLX-RAD treated animals relative to the untreated irradiated mice to reach a similar effect.

The PLX-RAD cells were shown to express in vitro several possible factors which may affect the BM. These factors include IL-6 IL-8 and IL-10 [61]–[68]. FGF-7 and MCP-3 [69]–[71]. But it is expected that the profile of cytokines secretion in vitro may be only a fraction of their potential in-vivo secretion profile when these cells are exposed to systemic messages from the radiation induced BM stress.

Another possible mechanism of action of PLX-RAD treatment may be also associated with the support of the regeneration of the radiation damaged BM niche. These supporting tissues establish a balance between self-renewal and differentiation of HSC [32], [49], [60]. PLX cells were previously shown to be able to induce tissues angiogenesis and revascularization, with relevant clinical application for critical limb ischemia [72]–[80]. Vascular stabilizing factors may also contribute to the protection of the BM niche following irradiation to improve the survival of the affected HSC after exposure to high dose of radiation [52]. We expect that further studies on the secretion of modulating factors by the PLX-RAD may provide the mechanism of action for these cells.

It was previously suggested that other irradiated organs such as the gastrointestinal tract may contribute to the damage of hematopoietic system in total body irradiation [81], [82]. The possible protective effect of PLX-RAD on other systems, such as the intestine may also contribute to the recovery of the BM failure in acute radiation syndrome. This point deserves further investigation.

### Conclusions

In conclusion, our data show the vast systemic effect of the placental derived PLX-mat and the optimal PLX-RAD in mitigating high dose lethal radiation damage. Such cells could be stored and delivered frozen, to be defrosted in the site of interest just before their injection. The PLX-RAD cells yield optimal effect even when first delivered by simple non-hazardous IM injections 1 or 2 days after a high dose irradiation with a 2^nd^ cell administration on day 5. The results suggest that the effects of different PLX based cell preparation are associated with the indirect enhancement of the residual BM HSC proliferation. The PLX-RAD cells rescued almost all the irradiated animals when treatment was initiated 24 or 48 hrs after irradiation. Further studies are conducted to explore the detailed mechanism and cascade of events behind the vast radioprotection by PLX-RAD cells. It is expected that the treatment may also have major applications for clinical conditions associated with severe BM aplasia.
